
JOURNAL INFORMATION
==============================
NLM Title Abbreviation: ScientificWorldJournal
Journal ID: ScientificWorldJournal
NLM Journal ID: 101131163
Journal ID: TSWJ

The Scientific World Journal

ISSN: 2356-6140
EISSN: 1537-744X
Publisher: Wiley

ARTICLE INFORMATION
==============================
PMCID: PMC5974846
PMID: 14570985
DOI: 10.1100/tsw.2003.74
Article ID: 747836
Article version: 1
Subjects: Editorial

Editorial — A New Method for Generic Measuring of the Global Quality of Life

Ventegodt Soren 1 *
Merrick Joav 2
Andersen Niels Jorgen 3
The Quality of Life Research Center, Teglgårdstræde 4-8, DK-1452 Copenhagen K, Denmark
National Institute of Child Health and Human Development, Ministry of Social Affairs, Jerusalem, Israel
Norwegian School of Management, Sandvika, Norway
*Soren Ventegodt: ventegodt@livskvalitet.org
Publication date: 2003
Electronic publication date: 2003 Oct 13
Volume: 3
First page: 946
Last page: 949
Copyright: Copyright © 2003 Soren Ventegodt et al.
Copyright year: 2003
License: This is an open access article distributed under the Creative Commons Attribution License, which permits unrestricted use, distribution, and reproduction in any medium, provided the original work is properly cited.
License URL: https://creativecommons.org/licenses/by/3.0/

==============================
Editorial
*TheScientificWorldJOURNAL* (2003) 3, 946-949
ISSN 1537-744X; DOI 10.1100/tsw.2003.74

### Editorial — A New Method for Generic Measuring of the Global Quality of Life

Søren Ventegodt[1,*], Joav Merrick[2], and Niels Jørgen Andersen[3]

[1]*The Quality of Life Research Center, Teglgårdstræde 4-8, DK-1452 Copenhagen K, Denmark;* [2]*National Institute of Child Health and Human Development, Office of the Medical Director, Division for Mental Retardation, Ministry of Social Affairs, Jerusalem and Zusman Child Development Center, Division of Community Health, Ben Gurion University, Beer-Sheva, Israel;* [3]*Norwegian School of Management, Sandvika, Norway*

**E-mail**: ventegodt@livskvalitet.org

*Received July 25, 2003; Revised August 15, 2003; Accepted August 16, 2003; Published October 13, 2003*

In association with the Danish Quality of Life Survey of 10,000 residents in Denmark, theories and research methods were developed through the last decade by the Quality of Life Research Center in Copenhagen. The methodological development peaked in 1990–1994 due to serious funding and a fruitful cooperation with the University Hospital of Copenhagen (Rigshospitalet)[1,2,3]. The discussions and research have resulted in the development of new generic questionnaires for generic measuring of the global quality of life (QOL), like SCREENQOL, SEQOL, and QOL5[4,5]. Since 1994, the scientific value of these methods and the SEQOL questionnaire have been subject to intense debate in Denmark, and to a much lesser extent also in Norway and Sweden. SEQOL and QOL5 have now been tested in practical use and recently have been validated statistically[4,5] and the QOL5 questionnaire received a prize given by the Danish Society for Vascular Surgery[4].

With this editorial, we launch a series of papers presenting the QOL measuring methodology. The papers present different aspects of this new methodology, and provide guidance on how the reader can use the SEQOL and QOL5 questionnaires in his or her own research.

The fundamental aim of the Danish Quality of Life Survey was to get quantitative data of a sufficiently high quality in order to test a possible causal connection between low quality of life and development of disease. The hypothesis to be tested is based on the assumption that we believe many illnesses of modern man are actually a consequence of low quality of life. We think that when quality of life is low, immune resistance is down, cellular order is disturbed, personal resources limited, talent difficult to draw on, creativity and performance poor, and personal development set on hold. If the hypothesis is right, then improving quality of life can prevent illness and illness may even be treated this way.

To test such a hypothesis in a prospective study following 10,000 persons from birth to death, we needed trustworthy quality-of-life figures on all population groups (i.e., generic measures) and a trustworthy and precise measure of the absolute, total quality of life (i.e., global measure) for each group. We also needed a questionnaire that was so thorough that we could assess the quality of life for a single person with some precision. A completely new strategy for measuring the global quality of life had to be developed, along with completely new questionnaires, as we could not find such questionnaires on the market.

*Corresponding author.
©2003 with author.

The articles in this series will not contain the quality-of-life philosophy behind the survey, the quality-of-life theories applied in the survey, or the results of the Quality of Life Survey (except for a few results that demonstrate the technical qualities of the survey), but this information will follow in other series of papers. Since 1995, we have also developed a similar technology for measuring the global quality of working life (QWL), complete with QWL philosophy, QWL theory, and the QWL questionnaire SEQWL. This again will be published in its own series of QWL papers.

The new technology in *generic measuring of the global quality of life* has been developed with generous support of more than 2 million dollars from many different foundations and sponsors. It is the result of the work of many brilliant people. One person in particular needs to be mentioned, the initiator of the project, the late pediatric professor Bengt Zachau-Christiansen, MD, PhD, MPH (1927–1997). Zachau-Christiansen started the Danish Quality of Life Survey as a continuation of his work with the Copenhagen Perinatal Birth Cohort 1959–1961[6,7] dating back to the 1950s, when work started on this unique study of child health and development. For the next 20 years, he directed the Perinatal Cohort, and in 1980 he asked the pediatrician Joav Merrick to perform that task[8]. When Joav Merrick moved to Israel in 1989 to become the Medical Director of the Ministry of Social Affairs and establish his own research center, Søren Ventegodt (at that time a medical student deeply involved in quality-of-life research) was asked to take charge of the project. Since the year 2000, Niels Jørgen Andersen from the Norwegian School of Management has participated in the research.

We hope that the ideas and techniques about measuring the global quality of life developed first by a study group at The University of Copenhagen[1,2,3] and since 1994 developed by the Quality of Life Research Center in Copenhagen, will inspire a wider audience. We are also very happy with this collaboration between the research centers in Denmark, Norway, and Israel, as an example of a fruitful international cooperation in quality-of-life research. If our culture is to develop positively, the society in which we live must emphasize more the potential of the individual in order to improve his or her life.

### CAN THE QUALITY OF LIFE BE MEASURED?

Typically, practical research on the quality of life attempts to measure (rate) the quality of life of a group of people. But is this not a contradiction? The quantity of life can be measured, as for example: how many people are alive 10 years after contracting a specific type of cancer, how many miscarriages have occurred, or how many kilograms a person weighs above the average. But the quality of life is a qualitative entity, so maybe it is not possible to measure?

Researchers can determine the quality of life of a group of people using a qualitative approach. One might conduct semi-structured interviews with people, observe them in their everyday lives, give them realistic problems to solve, ask them to daydream, play role games, or make them undertake other exercises that reveal the depths of their inner being. Such an approach will allow subjects to express their own views on the quality of life in a nonquantitative manner. Such close and sensitive descriptions of life are important and may result in better treatment or a change in healthcare priorities. This is especially true for life-altering surgical interventions such as the removal of a cancer or an organ transplant.

Unfortunately, these qualitative methods are problematic and also time and fund consuming. A researcher may inadvertently bias the data as a result of preconceived theories, prejudices, and notions. It is easy to overlook factors that contradict one's beliefs. If a researcher who is a staunch nonsmoker surveys the quality of life of smokers, the deep-rooted notion that the quality of life of smokers is bound to be poorer in the long run may affect the researcher during interviews. In fact, the quality of life of smokers might not vary significantly from that of nonsmokers (as in fact our Quality of Life Survey concluded).

The results of all studies, including quantitative surveys, are influenced by the minds and attitudes of the researchers conducting them. However, it may be far more difficult to argue against numbers reached through measurement. Although the manner in which the ratings are reached is full of problems and pitfalls (some of which are dealt with in subsequent papers), scores tend to have great dynamic force by virtue of their simplicity. Although numbers are not objective, they are outside the purely private sphere and thereby provide a new, collective, and sober reconsideration of the circumstances they represent.

The present survey was conducted in this light: not with the aim of establishing final truths on the quality of life of people under given circumstances, but to indicate relationships and to inspire people to reflect, talk, and start to discuss these matters.

Quantitative and qualitative studies should move hand in hand. The insights and intuitive realizations attained qualitatively ought to be tested quantitatively. These surveys may be surprising in what they reveal and have to be interpreted and illustrated qualitatively. This gain should lead to new, quantitative checks. This is the dialectic process *par excellence*.

The series of papers soon to appear will present further considerations about quality-of-life research methodology. We plan to present the following papers for the scientific community:

- Measuring the Quality of Life I. A Methodological Framework
- Measuring the Quality of Life II. From the Philosophy of Life to Science
- Measuring the Quality of Life III. From the IQOL Theory to the Global, Generic SEQOL Questionnaire
- Measuring the Quality of Life IV. Use of the SEQOL, QOL5, QOL1, and Other Global and Generic QOL Questionnaires
- Measuring the Quality of Life V. How to Use the SEQOL, QOL5, and QOL1 and Other Global and Generic QOL Questionnaires for Research
- Measuring the Quality of Life VI. Quality-Adjusted Life Years (QALY) is An Unfortunate Use of the Quality-of-Life Concept.
- Measurement of Quality of Life VII. Statistical Covariation and Global Quality-of-Life Data: The Method of Weight-Modified Linear Regression

### REFERENCES

1. Ventegodt, S. (1991) What is quality of life? Discussion in the "Project Group for Quality of Life Research in Copenhagen". *Ugeskr. Læger* **153**, 2201–2202. (Danish)
2. Ventegodt, S., Poulsen, D.L., and Hilden, J. (1992) Five theories about quality of life. Results from one year's work of the "Project Group for Quality of Life Research in Copenhagen". *Ugeskr. Læger* **154**, 585–586. (Danish)
3. Ventegodt, S., Poulsen, D.L., Hilden, J., Frimodt, V., Lohmann-Devantier, E., and Pruzan P. (1994) New tools to measure quality of life. *Agrippa* **14(3–4)**, 210–215. (Danish)
4. Lindholt, J.S., Ventegodt, S., and Henneberg, E.W. (2002) Development and validation of QOL5 for clinical databases. A short, global and generic questionnaire based on an integrated theory of the quality of life. *Eur. J. Surg.* **168,** 107–103.
5. Ventegodt, S., Henneberg, E.W., Merrick, J., and Lindholt, J.S. (2003) Validation of two global and generic quality of life questionnaires for population screening: SCREENQOL & SEQOL. *TheScientificWorldJOURNAL* **3**, 412–421.
6. Zachau-Christiansen, B. (1972) The influence of prenatal and perinatal factors on development during the first year of life with special reference to the development of signs of cerebral dysfunction. A prospective study of 9,006 pregnancies. Poul A. Andersen, Elsinore.
7. Zachau-Christiansen, B. and Ross, E.M. (1975) *Babies*: *Human Development During the First Year*. John Wiley & Sons, London.
8. Merrick, J., Merrick, Y., and Zachau-Christiansen, B. (1983) The Copenhagen Perinatal Cohort 1959–61. *Int. J. Rehab. Res.* **6(1)**, 57–59.

**This article should be referenced as follows**:

Ventegodt, S., Merrick, J., and Anderson, N.J. (2003) Editorial — a new method for generic measuring of the global quality of life. *TheScientificWorldJOURNAL* **3**, 946–949.

### BIOSKETCHES

**Søren Ventegodt, MD,** is the Director of the Quality of Life Research Center in Copenhagen, Denmark. He is also responsible for a Research Clinic for Holistic Medicine in Copenhagen and is a popular speaker throughout Scandinavia. He has published numerous scientific or popular articles and a number of books on holistic medicine, quality of life, and quality of working life. His most important scientific contributions are the comprehensive SEQOL questionnaire, the very short QOL5 questionnaire, the integrated quality-of-life (IQOL) theory, the holistic process theory, the life mission theory, and the Danish Quality of Life Research Survey, 1991–94 in cooperation with the University Hospital of Copenhagen and the late pediatric professor Bengt Zachau-Christiansen. E-mail: ventegodt@livskvalitet.org. Website: www.livskvalitet.org/

**Joav Merrick, MD, DMSc,** is Professor of Child Health and Human Development affiliated with the Zusman Child Development Center and Division of Community Health at the Ben Gurion University, Beer-Sheva, Israel and presently the Medical Director of the Division for Mental Retardation, Ministry of Social Affairs, Jerusalem and the Director of the National Institute of Child Health and Human Development. He has numerous publications in the field of child and human development, rehabilitation, intellectual disability, disability, health, welfare, abuse, advocacy and prevention. Dr. Merrick received the Peter Sabroe Child Award for outstanding work on behalf of Danish Children in 1985 and the International LEGO-Prize ("The Children's Nobel Prize") for an extraordinary contribution towards improvement in child welfare and well being in 1987. E-mail: jmerrick@internet-zahav.net. Website: www.nichd-israel.com

**Niels Jørgen Andersen, MSc,** Professor, Department of Innovation and Economic Organization, Norwegian School of Management. This department conducts research and provides teaching in central topics related to innovation, business development, management of global companies, business history, and economic organization. Research activities within the Department are related to four core subjects within the discipline: business history, cooperative organizations, business development and entrepreneurship, and finally studies of industries with a special focus on the electricity industry. He is also the dynamic chairman of the nonprofit organization Stiftelsen Holistisk Medisin Scandinavia, which aims to support the scientific development, research, and documentation of complementary and holistic medicine in Scandinavia. E-mail: niels.j.andersen@bi.no. Website: www.bi.no/users/fgl93013/
